# 1352. A Precision Medicine Approach to Predicting “Long COVID” through Machine Learning Analysis of Whole Blood Transcriptome Data

**DOI:** 10.1093/ofid/ofad500.1189

**Published:** 2023-11-27

**Authors:** Nusrat J Epsi, Stephanie A Richard, Josh Chenoweth, David Lindholm, Katrin Mende, Anuradha Ganesan, Nikhil Huprikar, Tahaniyat Lalani, Alfred Smith, Rupal Mody, Christina Schofield, Rhonda E Colombo, Ryan Flanagan, Evan Ewers, Catherine Berjohn, David Saunders, Milissa Jones, Margaret Edwards, Mark P Simons, Robert O’Connell, David R Tribble, David R Tribble, Brian Agan, Timothy Burgess, Clifton Dalgard, Simon Pollett

**Affiliations:** Uniformed Services University of the Health Sciences, Bethesda, Maryland; Infectious Disease Clinical Research Program, Department of Preventive Medicine and Biostatistics, Uniformed Services University of the Health Sciences, Bethesda, MD, USA, Bethesda, Maryland; Henry M. Jackson Foundation, Bethesda, Maryland; Department of Medicine, Uniformed Services University of the Health Sciences; Brooke Army Medical Center, San Antonio, Texas; Brooke Army Medical Center, San Antonio, Texas; Infectious Disease Clinical Research Program, USUHS; Henry M. Jackson Foundation for the Advancement of Military Medicine Inc, Bethesda, Maryland; Walter Reed National Military Medical Center, Bethesda, Maryland; Naval Medical Center Portsmouth, Portsmouth, Virginia; Naval Medical Center, Portsmouth, Virginia; William Beaumont Army Medical Center, El Paso, Texas; Madigan Army Medical Center, Tacoma, Washington; Infectious Disease Clinical Research Program, USUHS; Henry M. Jackson Foundation for the Advancement of Military Medicine, Inc., Bethesda, Maryland; Tripler Army Medical Center, Honolulu, Hawaii; Fort Belvoir Community Hospital, Fort Belvoir, Virginia; Naval Medical Center San Diego, San Diego, California; Uniformed Services University of the Health Sciences, Bethesda, MD, USA, Bethesda, Maryland; Uniformed Services University of the Health Sciences, Bethesda, Maryland; Henry M Jackson Foundation for the Advancement of Military Medicine, bethesda, Maryland; Infectious Disease Clinical Research Program, Department of Preventive Medicine and Biostatistics, Uniformed Services University of the Health Sciences, Bethesda, MD, USA, Bethesda, Maryland; Infectious Disease Clinical Research Program, USUHS, Bethesda, Maryland; Uniformed Services University of the Health Sciences, Bethesda, Maryland; Uniformed Services University of the Health Sciences, Bethesda, Maryland; Infectious Disease Clinical Research Program, Department of Preventive Medicine and Biostatistics, Uniformed Services University of the Health Sciences, Bethesda, MD, USA, Bethesda, Maryland; Infectious Disease Clinical Research Program, Department of Preventive Medicine and Biostatistics, Uniformed Services University of the Health Sciences, Bethesda, MD, USA, Bethesda, Maryland; The American Genome Center, Uniformed Services University of the Health Sciences, Bethesda, Maryland; Infectious Disease Clinical Research Program, Department of Preventive Medicine and Biostatistics, Uniformed Services University of the Health Sciences, Bethesda, MD, USA, Bethesda, Maryland

## Abstract

**Background:**

Predicting, preventing, and treating post-COVID condition (PCC; “Long COVID) is challenging due to a limited understanding of PCC mechanisms. To address this, we analyzed whole blood transcriptomic data using machine learning to identify candidate gene and gene expression pathways associated with the development of PCC.

**Figure 1**

**Figure:**
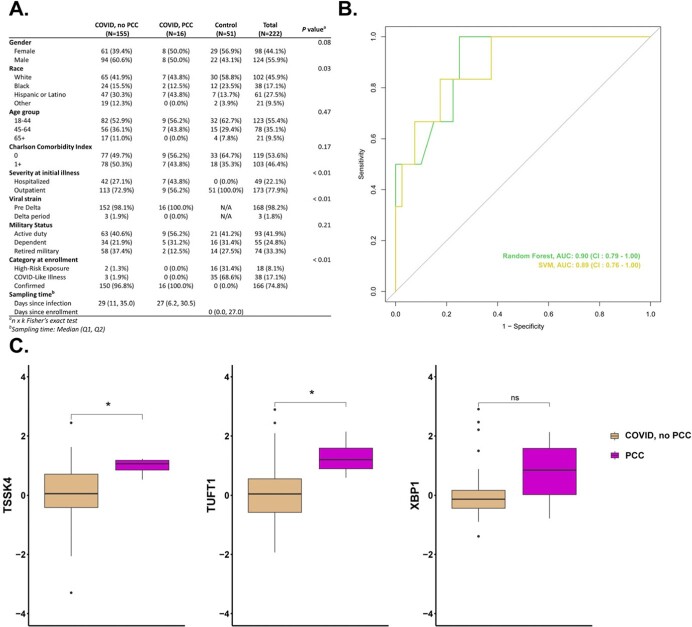

(A) Clinical and demographic characteristics of 222 military health system beneficiaries (COVI D, no PCC: SARS-CoV-2 positive subjects reported no or mild symptoms on 6-month survey; COVID, PCC: SARS-CoV-2 positive subjects reported moderate to severe symptoms on 6-month survey; and Control: SARS-CoV-2 negative subjects), (B) AUROC indicates the ability of leading genes (measured early after infection, TSSK4, TUFT1, and XBP1; which have known association with pulmonary fibrosis, lung cancer, and lymphoma, respectively) which can predict which participants develop PCC versus those who do not, and (C) Identification of biomarker candidates for PCC based on machine learning algorithms. Statistical significance was determined by Mann-Whitney U test. Asterisks indicate statistical significance: ns: p > 0.05, *: p ≤ 0.05

**Methods:**

The Epidemiology, Immunology, and Clinical Characteristics of Emerging Infectious Diseases with Pandemic Potential (EPICC) study is a longitudinal cohort study exploring the impact of SARS-CoV-2 infection in military health system beneficiaries. We collected demographic and clinical data through surveys, interviews and medical record reviews. We conducted transcriptome profiling (bulk RNA-seq) on early post-infection whole blood samples from unvaccinated individuals with and without PCC at 6-months, as well as uninfected, unvaccinated controls. These three groups were matched on sex, age, and comorbidities. We identified transcriptomic signatures associated with group classes using t-tests with correction for multiple comparisons. Molecular signatures and candidate markers for PCC were developed using gene set enrichment analysis (GSEA) and machine learning, including random forest (RF) and support vector machine (SVM) methods.

**Results:**

Out of the 5289 SARS-CoV-2 positive Military Health System beneficiaries enrolled in EPICC, 814 participants had at least one sample with blood transcriptomic profiling data. Among these, 171 unvaccinated, SARS-CoV-2 positive participants completed a 6-month follow up survey and were included in the analysis. Ninety-one percent did not report any chronic symptoms, while 8% reported moderate to severe symptoms at six months and were classified as PCC group. A control group of 51 participants was also identified. Machine learning approaches (RF: AUROC = 0.90, CI = 0.79 to 1; and SVM: AUROC = 0.89, CI = 0.76 to 1) identified three candidate biomarkers (Figure 1), including TSSK4, TUFT1, and XBP1 genes, which could be a predictive or mechanistic marker for PCC with further validation.

**Conclusion:**

Our study demonstrates the potential of using machine learning to translate transcriptomic data into precision medicine applications, specifically for predicting the development of PCC after COVID-19.

**Disclosures:**

**Mark P. Simons, PhD**, AstraZeneca: The IDCRP and HJF were funded to conduct an unrelated phase III COVID-19 monoclonal antibody immunoprophylaxis trial as part of US Govt COVID Response **Timothy Burgess, MD, MPH**, AstraZeneca: The IDCRP and the Henry M. Jackson Foundation (HJF) were funded to conduct an unrelated phase III COVID-19 monoclonal antibody immunoprophylaxis trial **Simon Pollett, MBBS**, AstraZeneca: The IDCRP and the Henry M. Jackson Foundation (HJF) were funded to conduct an unrelated phase III COVID-19 monoclonal antibody immunoprophylaxis trial

